# Stress-Related Neuronal Clusters in Sublenticular Extended Amygdala of Basal Forebrain Show Individual Differences of Positions

**DOI:** 10.3389/fncir.2020.00029

**Published:** 2020-05-28

**Authors:** Munenori Kanemoto, Tomoya Nakamura, Masakiyo Sasahara, Hiroyuki Ichijo

**Affiliations:** ^1^Department of Anatomy and Neuroscience, Faculty of Medicine, University of Toyama, Toyama, Japan; ^2^Department of Pathology, Faculty of Medicine, University of Toyama, Toyama, Japan

**Keywords:** basal forebrain, extended amygdala, zif268/egr1, c-fos, GABAergic, restraint stress, asymmetry, individual difference

## Abstract

To understand functional neuronal circuits for emotion in the basal forebrain, patterns of neuronal activation were examined in mice by immunohistochemistry of immediate-early gene products (Zif268/Egr1 and c-Fos). In all mice examined, clusters of 30–50 neurons expressing Zif268 were found on both sides in the area between the extended amygdala (EA) and globus pallidus (GP), generally designated as sublenticular extended amygdala (SLEA). The clusters consisted of 79.9 ± 3.0% of GABAergic neurons in GAD65-mCherry mice. The expression of the cholinergic marker choline acetyltransferase and the GP markers parvalbumin, proenkephalin, and FoxP2 indicated that these neurons were different from known types of neurons in the EA and GP; therefore, we named them the sublenticular extended amygdalar Zif268/Egr1-expressing neuronal cluster (SLEA-zNC). Sublenticular extended amygdalar Zif268/Egr1-expressing neuronal clusters participated in stress processing because increasing numbers of cells were observed in SLEA-zNCs after exposure to restraint stress (RS), the induction of which was suppressed by diazepam treatment. Mapping SLEA-zNCs showed that their positions and arrangement varied individually; SLEA-zNCs were distributed asymmetrically and tended to be situated mainly in the middle region between the anterior commissure (AC) and posterior end of the GP. However, the total cell number in SLEA-zNCs was compatible between the right and left hemispheres after activation by RS. Therefore, SLEA-zNCs were distributed asymmetrically but were not lateralized. Because time courses of activation differed between the Zif268 and c-Fos, the sequential dual treatment of RSs enabled us to differentiate SLEA-zNCs activated by the first and second RS. The results supported that the same SLEA-zNCs responded to both the first and second RS, and this also applied for all SLEA-zNCs. Thus, we concluded that the cluster positions were invariable under RS in each mouse but were distributed differently between individual mice. We name these newly identified neuronal clusters as stress-related neuronal clusters, SLEA-zNCs, which are considered to be novel functional units of “islands of activation.” Moreover, SLEA-zNCs were situated at different positions in all mice examined, showing individual differences in their positions.

## Introduction

The basal forebrain (BF) is composed of various structures including the extended amygdala (EA) and peripallidal regions (Mesulam et al., 1983; Masuda et al., 1997), which have been implicated in cortical activation, as well as facilitating processes of motivation, attention, learning, and memory (Conner et al., 2003; Jones, 2008; Parikh and Sarter, 2008; Goard and Dan, 2009). A part of the EA was formerly called the substantia innominata (SI). The rostral region of the SI is mainly composed of the nucleus accumbens and the ventral extensions of the globus pallidus (GP). The caudal region of the SI comprised the EA, which refers to the regions encompassing the centromedial amygdala and the bed nucleus of the stria terminalis (de Olmos and Heimer, 1999; Riedel et al., 2002; Zaborszky et al., 2012). The BF contains heterogeneous cell types that are cholinergic, γ-aminobutyric acid (GABA)-ergic, glutamatergic projection neurons, and local GABAergic/peptidergic interneurons in rodents and primates (Duque et al., 2000; Gritti et al., 2006; Heimer et al., 2008; Zaborszky et al., 2015).

The calcium-binding proteins parvalbumin (PV) and calbindin D-28k (CB) are useful for distinguishing subgroups of GABAergic neurons neurochemically (Kemppainen and Pitkänen, 2000; Gritti et al., 2003). Distribution of PV- and CB-positive cells differentiates the EA from other structures of the BF (Hontanilla et al., 1998; Zahm et al., 2003). In the GP, GABAergic neurons are classified into prototypic neurons, which express mostly PV, and arkypallidal neurons, which express exclusively the neuropeptide precursor proenkephalin (PENK) and the transcription factor FoxP2 (Mallet et al., 2012; Abdi et al., 2015; Dodson et al., 2015; Hernández et al., 2015).

Various methods have been used to obtain functional information about neuronal circuits. One method is the analysis of spatiotemporal patterns of immediate-early gene (IEG) expression, which is transiently up-regulated in neurons by various stimuli. The expression level of IEGs is a reliable marker for increasing neuronal activity, and the expression of different IEGs has different stimulus thresholds for transcriptional induction (Guzowski et al., 2001; Zangenehpour and Chaudhuri, 2002; Gallo et al., 2018). Thus, neuronal expression of IEGs after stimulation indicates spatiotemporal patterns of neuronal activation, which provide functional information about neuronal circuits. For example, neuronal circuits involved in stress processing have been examined with IEG expression. The expression of different IEGs is involved in region-specific functions (Ishida et al., 2000); the expression of one of the IEGs, Zif268/Egr1, is induced after stimulation of systemic stress (Cullinan et al., 1995; Senba and Ueyama, 1997; Ábrahám and Kovács, 2000). However, the anatomical complexity of the BF makes it difficult to differentiate the neuronal characteristics in the region because the EA and GP are intricately intertwined in their boundary area.

Here, we found stress-related clusters of neurons expressing Zif268 on both sides of the sublenticular extended amygdala (SLEA) between the EA and GP, which we named Zif268/Egr1-expressing neuronal cluster in SLEA, or sublenticular extended amygdalar Zif268/Egr1-expressing neuronal cluster (SLEA-zNC). These clusters are thought to be novel functional units of “islands of activation.” In addition, SLEA-zNCs were situated at diverse positions in all mice examined, showing individual differences of their positions.

## Materials and Methods

### Animals

C57BL/6J mice were obtained from Japan SLC (Hamamatsu, Shizuoka, Japan). GAD65-Cre mice (Gad2 < tm2(cre)Zjh > /J; JAX #010802) were obtained from The Jackson Laboratory (Bar Harbor, ME, United States) and were bred as homozygotes. The GAD65-mCherry mice were produced by crossing male Gad2-IRES-Cre knock-in mice and female mCherry Cre-reporter mice (R26R-H2B-mCherry; CDB0204K, Abe et al., 2011). The Cre activity in the Gad2-IRES-Cre knock-in mice has been reported as quite effective, activating the reporter allele in greater than 90% of the targeted cell populations in the cortex and hippocampus (Taniguchi et al., 2011). ROSA26 locus in the R26R-H2B-mCherry mice is an endogenous locus ubiquitously active (Abe et al., 2011). Therefore, high efficiency of expression is also expected in GABAergic neurons outside cortex and hippocampus. In the GAD65-mCherry mice, GAD65-positive neurons express histone H2B-mCherry fusion protein, which localizes in nuclei. Therefore, GAD65-positive GABAergic neurons were visualized by nuclear localization of mCherry fluorescence. Because, in this study, the GAD65-mCherry mice were not congenic and their genetic backgrounds were not identical, the analysis was confirmed in GAD67-GFP knock-in mice, which are congenic on the C57/BL6 background (RBRC03674, strain name: ICR.Cg-Gad1 <tm1.1Tama>) (Tamamaki et al., 2003). All mice were in a temperature-controlled room (23 ± 1°C) on a 12:12-h light–dark cycle (lights were turned on at 05:00 and off at 17:00). Mice were maintained in single-sex groups with up to five littermates per cage. Food (CE-2; CLEA Japan, Inc., Tokyo, Japan) and water were supplied *ad libitum*. Experiments were performed on 2- to 3-month-old mice. All mice used in the experiments with restraint stress (RS) and their controls were male. In the experiments for immunohistochemical characterization, five mice were used for each of the analyses; those were three males and two females. Because the immunohistochemical results were similar when males and females were analyzed separately, we pooled the results and presented the data of both males and females together.

### Immunohistochemistry

Mice were deeply anesthetized with an intraperitoneal injection of pentobarbital sodium (50 mg/kg body weight; Kyoritsu Seiyaku, Co., Tokyo, Japan) and transcardially perfused with phosphate-buffered saline (PBS) and 3.7% formaldehyde (#064-00406; Wako Pure Chemical Industries, Ltd., Osaka, Japan) in PBS. All mice were perfusion-fixed between 11:00 and 13:00 to reduce the effects of circadian variations. Their brains were immersed in 3.7% formaldehyde in PBS at 4°C overnight, embedded in gelatin (16.7% gelatin, 16.7% glycerol in PBS), and postfixed in 3.7% formaldehyde in PBS for 4 days at 4°C. The brains were coronally sectioned into 70-μm slices with a vibratome (Leica VT1000S; Leica Microsystems Nussloch, GmbH, Nussloch, Germany). The sections were rinsed in PBS with 0.5% Triton X-100 (#169-21105; Wako) (PBT) and blocked with 5% bovine serum albumin (#9048-46-8; Wako) in PBT. The following antibodies were used: anti-Zif268/Egr1 (rabbit polyclonal; 1:500; #sc-189; Santa Cruz Biotechnology, Inc., Santa Cruz, CA, United States), anti-c-Fos (goat polyclonal; 1:1,000; #sc-52-G; Santa Cruz), anti-NeuN (mouse monoclonal; 1:1500; #MAB377; Millipore, Co., Burlington, MA, United States), anti-GFAP (goat polyclonal; 1:1,000; #ab53554; Abcam, Inc., Cambridge, United Kingdom), anti-calbindin D-28k (goat polyclonal; 1:3,000; #C9848; Sigma-Aldrich, Co., St. Louis, MO, United States), anti-PV (mouse monoclonal; 1:9,000; #P3088; Sigma-Aldrich), anti-PENK (goat polyclonal; 1:500; #OAEB00439; Aviva Systems Biology, Co., San Diego, CA, United States), anti-FoxP2 (goat polyclonal; 1:1,000; #ab1307; Abcam, Inc.), and anti-choline acetyltransferase (ChAT) (goat polyclonal; 1:1,000; #AB144P; Millipore, Co.). Double immunohistochemical stainings were visualized using the following fluorescent secondary antibodies: donkey anti-rabbit Alexa Fluor 488 (1:400; #A21206; Invitrogen, Co., Carlsbad, CA, USA), donkey anti-rabbit Alexa Fluor 594 (1:400; #A21207; Invitrogen), donkey anti-mouse Alexa Fluor 488 (1:400; #A21202; Invitrogen, Co.), donkey anti-goat Alexa Fluor 488 (1:400; #A11055; Invitrogen, Co.), and donkey anti-goat Alexa Fluor 647 (1:400; #AP180SA6; Millipore, Co.). Single staining was visualized using the avidin-biotin complex (ABC) method (Vectastain Elite ABC kit; #PK6100; Vector Laboratories, Inc., Burlingame, CA, United States) with the nickel-enhanced 3.3′-diaminobenzidine (DAB) system (Pierce Metal Enhanced DAB Substrate Kit; #34065; Thermo Fisher Scientific, Inc., Lake Barrington, IL, United States). Sections were counterstained with NeuroTrace 500/525 green fluorescent Nissl stain (1:400; #N21480; Invitrogen, Co.) or 4′,6-diamidino-2-phenylindole (DAPI, 1:10,000; #D9542; Sigma-Aldrich), mounted on glass slides with Mowiol 4-88 reagent (#475904; Calbiochem-Novabiochem, Co., San Diego, CA, United States), observed under a fluorescence microscope (DMRE TCS SP2, Leica), and photographed with a digital camera (camera head: DS-Ri1; control unit: DS-U3; Nikon, Co., Tokyo, Japan). Confocal images of SLEA-zNCs were obtained in optically serial sections of 3-μm intervals with the LSM780 system (Carl Zeiss MicroImaging, GmbH, Jena, Germany). Images were processed using the ImageJ software (National Institutes of Health, Baltimore, MD, United States).^1^ Positive cells for each cell-type marker were counted in five mice.

### Mapping Anteroposterior Positions of SLEA-zNCs

**FIGURE 1:**
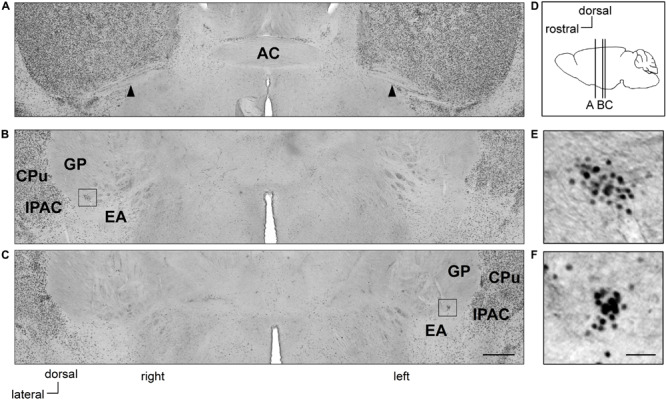
Clusters of Zif268-positive cells are situated asymmetrically in the sublenticular extended amygdala between the extended amygdala and globus pallidus. Coronal sections of mouse brains are shown in panels **(A–C)**. Positions of sections along the anteroposterior axis are indicated schematically at a lateral view of the mouse brain **(D)**. Clusters of Zif268-positive cells are shown in low magnification **(B,C)** and in high magnification **(E,F)**. A cluster of Zif268-positive cells is shown on the right hemisphere at 700 μm posterior to the AC **(B)**, another in the left hemisphere at 840 μm posterior to the AC **(C)**. Panels **(E,F)** are magnified views of the squares in panels **(B,C)**, respectively. Arrowheads indicate the posterior limbs of the AC **(A)**. AC, anterior commissure; CPu, caudate-putamen; EA, extended amygdala; GP, globus pallidus; IPAC, interstitial nucleus of the posterior limb of the anterior commissure. Scale bars, 500 μm **(A–C)** and 50 μm **(E,F)**.

Mouse brains were processed for Zif268 immunohistochemistry with the ABC-DAB system described in *Immunohistochemistry* above. By using series of serially cut coronal sections from the anterior commissure (AC) to the posterior GP, anteroposterior positions of SLEA-zNCs were mapped; distances were measured from the posterior limb of the AC to SLEA-zNCs. Because both SLEA-zNCs and the posterior limb of the AC were located in the same horizontal plane at approximately 4.5–5.0 mm from the dorsal surface of the brain, and in almost the same parasagittal plane at approximately 2 mm lateral from the midline (Figure 1; Paxinos and Franklin, 2013), it was possible to evaluate their anteroposterior distances accurately by counting the number of sections between them, which was not influenced by the tilt of the sections. The measurement of distance could only produce a possible error within the thickness of the sections (70 μm). To present data of the mapping (Figures 5A–D), when the coronal planes suffered from yawing (a tilt along the dorsoventral axis), their positions were corrected with reference to both sides of the posterior limb of the AC as a landmark of tilting. The entire length of the samples along the anteroposterior axis was divided into a series of 70-μm interval; the number of SLEA-zNCs that appeared in each interval was divided by the number of mice examined, obtaining the frequency of their appearance. The frequency distribution of SLEA-zNCs is mapped in Figures 5E,F; for example, a frequency of “1” meant that SLEA-zNCs were observed in all mice examined.

### Restraint Stress

Mice were singly housed a week before the experiment. In pilot experiments, SLEA-zNCs were observed in the brains of all mice (whether single-housed or in groups) fixed immediately from their home cages, and the numbers of neurons in SLEA-zNC were not significantly different between the home-caged (*n* = 5, male mice, 31.8 ± 3.4; data are expressed as mean ± standard error of the mean) and single-housed mice (*n* = 5, male mice, 40.2 ± 3.8) (Wilcoxon rank-sum test, *p* = 0.206). Thus, in this study, we used the single housing with no RS as the control, although single housing is known to be a stressor for rodents (Karolewicz and Paul, 2001). Mice were restrained in 50-mL plastic conical tubes (#339652; Thermo Fisher Scientific, Inc., Waltham, MA, United States) with 3-mm air holes for 60 min (RS). After RS, mice were singly transferred into a clean housing cage. To examine the expression of Zif268, mice were perfusion-fixed at 30, 60, 120, or 180 min after RS. In control animals (Ctrl), mice were taken directly from their housing cage without RS and were perfusion-fixed. Brains were processed for Zif268 and c-Fos double immunofluorescence.

As a positive control, the stress response in the paraventricular thalamic nucleus (PVT), which is known to be the stress-related neuronal nucleus, was evaluated (Bubser and Deutch, 1999; Otake et al., 2002). The numbers of Zif268-positive cells were counted in three serial sections of the middle region of the PVT.

### Corticosterone Measurement

Blood was sampled between 12:00 and 13:00. Immediately prior to perfusion, blood was collected in a 1.5-mL tube containing 2 U/mL of heparin sodium (5,000 units/5 mL; Mochida Pharmaceutical, Co., Tokyo, Japan) by puncturing the left ventricle (1/2 inch, 26-gauge needle; #NN-2613S; Terumo, Co., Tokyo, Japan). Samples were centrifuged at 3,000 × *g* for 10 min at 4°C. The plasma samples were stored at −80°C until the day of analysis. Plasma corticosterone was measured with AssayMax Corticosterone ELISA Kit (#EC3001-1; AssayPro, LLC, St. Charles, MO, United States). Each plasma sample (25 μL) and corticosterone standard sample were added into each well of a 96-well microplate precoated with a polyclonal antibody specific for corticosterone, and 25 μL of biotinylated corticosterone was added to each well. After a 2-h incubation, the wells were washed with wash buffer attached in the kit. Streptavidin–peroxidase (50 μL) was added and incubated for 30 min at room temperature. After washing with the wash buffer, 50 μL of chromogen substrate was added per well and incubated for 25 min. After adding 50 μL of stop solution, the absorbance was read immediately on a microplate reader (FilterMax F5; Molecular Devices, LLC, San Jose, CA, United States) at a wavelength of 450 nm. For each experiment, the mean values of the triplicate readings were calculated for each standard and sample. The experiments were repeated three times. The concentrations of corticosterone were determined from the standard curve.

### Diazepam Treatment

Diazepam (#045-18901; Wako) was dissolved in saline with 0.5% Tween-80 (#164-21591; Wako). A diazepam solution (0.5 mg/kg) or a vehicle solution (saline with 0.5% Tween-80 solution) was injected intraperitoneally in mice (Sugimoto et al., 1998; Gaier et al., 2010; Béracochéa et al., 2011). Mice were divided into four groups, as follows: (1) vehicle-Ctrl group, where 30 min after having received the vehicle solution, mice were perfusion-fixed; (2) diazepam-Ctrl group, where 30 min after having received the diazepam solution, mice were perfusion-fixed; (3) vehicle-RS group, where 30 min after having received the vehicle solution, mice were subjected to RS and then perfusion-fixed after 60 min after RS; and (4) diazepam-RS group, where 30 min after having received the diazepam solution, mice were subjected to RS and then perfusion-fixed 60 min after RS.

### Sequential Dual Treatment of Restraint Stresses

**FIGURE 2:**
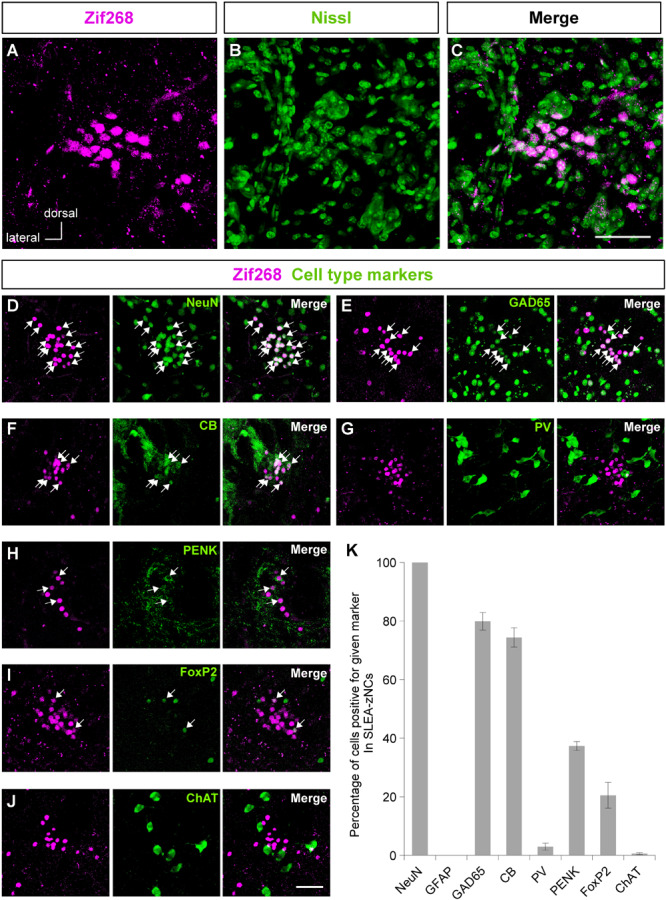
Sublenticular extended amygdalar Zif268/Egr1-expressing neuronal clusters (SLEA-zNC) are novel neuronal clusters. Zif268-positive cells in SLEA-zNCs [magenta in panel **(A)**] are not discernible by fluorescent Nissl staining [green in panel **(B)**]. Panels **(A,B)** are merged into panel **(C)**. The Zif268-positive cells are shown in magenta **(D–J)**. Cell-type markers: NeuN **(D)**, GAD65 reporter **(E)**, CB **(F)**, PV **(G)**, PENK **(H)**, FoxP2 **(I)**, and ChAT **(J)** are shown in green. Arrows indicate double-positive cells. The percentages of cells positive for a given marker in SLEA-zNCs are shown in panel **(K)**. Data are represented as mean ± SEM. SLEA-zNC, sublenticular extended amygdalar neuronal cluster; GFAP, glial fibrillary acidic protein; CB, calbindin D-28k; PV, parvalbumin; PENK, proenkephalin; ChAT, choline acetyltransferase. Scale bars, 50 μm **(A–J)**.

**FIGURE 3:**
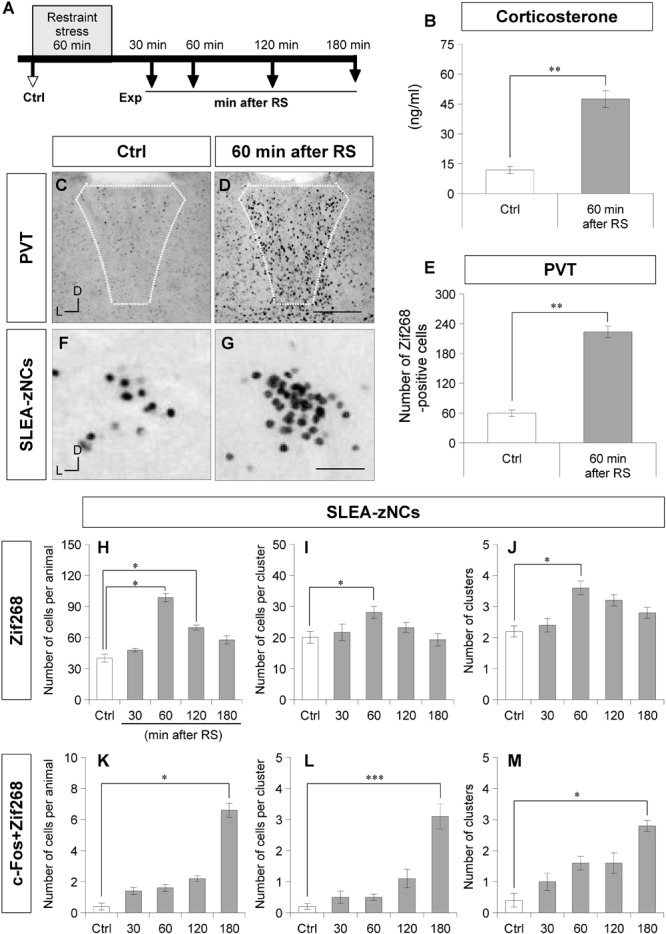
Sublenticular extended amygdalar Zif268/Egr1-expressing neuronal clusters are activated by restraint stress (RS). The time course of the stress experiment is shown **(A)**. Control (Ctrl) indicates no RS, and the experiments (Exp) indicate 30, 60, 120, or 180 min after RS. The plasma corticosterone concentrations are shown for the Ctrl group and the 60-min-after-RS group **(B)** (Wilcoxon rank-sum test, *n* = 6 animals per condition). Zif268-positive cells in the PVT are shown in a Ctrl animal **(C)** and a 60-min after RS animal **(D)**. White dotted lines indicate the boundary of the PVT. The numbers of Zif268-positive cells in the PVT are shown for the Ctrl and the 60-min after RS animals **(E)**. Wilcoxon rank-sum test, *n* = 6 animals per condition. The Zif268-positive cells in SLEA-zNCs are shown in a Ctrl **(F)** and a 60-min after RS animal **(G)**. The numbers of Zif268-positive cells in SLEA-zNCs per animal are shown in the Ctrl and at 30, 60, 120, and 180 min after RS **(H)**. The numbers of Zif268-positive cells in each SLEA-zNC **(I)**. The numbers of SLEA-zNCs **(J)**. The numbers of c-Fos + Zif268 double-positive cells in SLEA-zNCs per animal **(K)**. The numbers of double-positive cells in each SLEA-zNC **(L)**. The numbers of SLEA-zNCs containing double-positive cells **(M)** (*n* = 5 animals per condition in panels **H**,**J**,**K**, and **M**, and *n* = 10, 11, 18, 15, 12 clusters for the conditions from Ctrl to 180 min after RS, respectively, in panels **(I,L)**. From **(H–M)**, values are compared with the Kruskal–Wallis test followed by the Steel test. Data are represented as mean ± SEM. Single, double, and triple asterisks indicate significances (^∗^*p* < 0.05, ^∗∗^*p* < 0.01, ^∗∗∗^*p* < 0.001). D, dorsal; L, lateral; SLEA-zNC, sublenticular extended amygdalar Zif268-expressing neuronal cluster; PVT, paraventricular thalamic nucleus. Scale bars, 250 μm **(D,E)** and 50 μm **(F,G)**.

In this study, taking advantage of the protein expression of two IEGs: Zif268 and c-Fos, it was established that the method for analyzing the time course of neuronal activation immunohistochemically was that of sequential dual treatment of RSs (dRS). Dual treatment of RS consisted of the first RS for 60 min, no RS for 60 min in a clean housing cage, and the second RS for another 60 min. Mice were divided into four groups: (1) Ctrl, where mice were taken directly from their housing cage without RS and perfusion-fixed (Figure 6A); (2) 60 min after single RS (sRS), where mice were perfusion-fixed 60 min after being treated with a single-60 min RS (Figure 6A); (3) 180 min after sRS, where mice were perfusion-fixed 180 min after being treated with a single-60 min RS (Figure 6A); and (4) 60 min after dRS, where mice were perfusion-fixed 60 min after being treated with the second RS of dRS (Figure 6B). Mouse brains were processed for immunohistochemistry and double-stained with anti-Zif268 and anti-c-Fos antibodies. Sublenticular extended amygdalar Zif268/Egr1-expressing neuronal clusters were examined under a confocal microscope in 10 optically serial sections at 3-μm intervals. Because the neuronal nuclear size in SLEA-zNC was 9.8 ± 0.1 μm in diameter (*n* = 245 neurons from five mice) and the same nucleus was observed in approximately three consecutive sections, the positive nuclei were separated from one another, and the double-positive nuclei were clearly identified. These optical sections were divided into five anterior (anterior half) and five posterior (posterior half) sections.

### Statistical Analysis

Data are expressed as mean ± standard error of the mean (SEM). Statistical analysis of differences between two groups was performed using the Wilcoxon rank-sum test. Comparisons of multiple groups were analyzed using the Kruskal–Wallis test followed by the Steel test or Steel-Dwass test. *p* < 0.05 was considered statistically significant. All statistical analyses were carried out using the “R” software (version 3.5.2; The R Foundation for Statistical Computing, Vienna, Austria; ISBN 3-900051-07-0).^2^

## Results

### Clusters of Zif268-Positive Cells in SLEA, in the Right and Left Hemisphere

In Ctrl C57BL/6J mice with no RS treatment, clusters of Zif268-positive cells were observed on both sides of the SLEA; they were clearly distinguished from surrounding cells as islands of Zif268-positive cells, flanked dorsally to the EA and ventrally to the GP by few positive cells, and medially from the interstitial nucleus of the anterior commissure (IPAC) by a high density of positive cells. The clusters were found in all mice examined [4.8 ± 0.5 (mean ± standard error) clusters/mouse from nine Ctrl mice; 2.7 ± 0.3 and 2.1 ± 0.3 clusters/mouse in the right and left hemisphere, respectively]. In a representative case, one cluster was found on the right side, 700 μm posterior to the posterior limb of the AC; the other was located on the left side, 840 μm to the posterior limb of the AC (Figure 1). The overall spatial distribution of the clusters is described in detail in section “Distribution of SLEA-zNCs Between the Right and Left Hemispheres at Different Positions Along the Anteroposterior Axis in Individual Mice.”

The clusters of Zif268-positive cells were observed within a close proximity of 80- to 120-μm diameter; however, they were not defined by expression of another IEG c-Fos in resting condition with no stimulation. Each cluster consisted of 35.1 ± 2.7 cells (average of 24 clusters from 10 mice). The size of the Nissl-stained cell soma in the clusters was 12.6 ± 0.3 μm (average of 76 cells from three male mice). Fluorescent Nissl staining did not reveal a distinct cluster cytoarchitecture (Figures 2A–C). Expressions of cell-type markers showed that the Zif268-positive cells within the clusters expressed NeuN (100%, *n* = 10 clusters from five mice; Figure 2D) but not GFAP (0%, *n* = 10 clusters from five mice; not shown), showing that the clusters were exclusively composed of neurons. The clusters expressed the GAD65 reporter in GAD65-mCherry mice [79.9 ± 3.0%, *n* = 10 clusters from five mice (Figure 2E)] and GAD67 reporter in GAD67-GFP knock-in mice [48.3 ± 6.5%, n = 10 clusters from five mice (data not shown).] In addition, they expressed CB (74.4 ± 3.3%, *n* = 10 clusters from five mice), PV (3.1 ± 1.2%, *n* = 10 clusters from five mice), PENK (37.3 ± 1.5%, *n* = 10 clusters from five mice), FoxP2 (20.5 ± 4.4%, *n* = 10 clusters from five mice), or ChAT (0.6 ± 0.3%, *n* = 10 clusters from five mice) in C57BL/6J mice (Figures 2F–J). The clusters were in different positions, composed of similar proportion of heterogeneous cell types; they are considered to be novel groups of neurons. Therefore, the clusters were named the “Zif268/Egr1-expressing neuronal cluster in sublenticular extended amygdala, or sublenticular extended amygdalar Zif268/Egr1-expressing neuronal cluster (SLEA-zNC).” Sublenticular extended amygdalar Zif268/Egr1-expressing neuronal clusters were mainly composed of GABAergic neurons (Figure 2K) and surrounded by ChAT-positive neurons. Sublenticular extended amygdalar Zif268/Egr1-expressing neuronal clusters’ cellular sizes were similar to those of the small GABAergic neurons (Ono et al., 2005).

### Response of SLEA-zNCs to Restraint Stress

Because there are stress-related neurons in the EA and GP, the response of SLEA-zNCs was examined against systemic stress using RS (Figure 3A; Cullinan et al., 1995; Senba and Ueyama, 1997; Ábrahám and Kovács, 2000). To confirm the systemic effects of RS, plasma corticosterone concentration was measured. Plasma corticosterone concentration was significantly higher in animals of the 60-min-after-RS group than in Ctrl animals (Wilcoxon rank-sum test, *n* = 6 mice for each condition, *p* = 0.002; Figure 3B).

After RS, a large number of Zif268-positive cells were observed in the PVT, a well-characterized, stress-related neuronal nucleus (Figures 3C,D). To confirm the neuronal effects of RS, the number of Zif268-positive cells was counted in the PVT; the number was significantly higher in animals of the 60-min-after-RS group than in Ctrl animals (Wilcoxon rank-sum test, *n* = 6 mice per group, *p* = 0.002; Figure 3E). These data confirmed that RS induced systemic stress responses and activated neuronal circuits related to stress processing.

In SLEA-zNCs, a large number of Zif268-positive cells were observed after RS (Figures 3F,G). The number of Zif268-positive cells per animal was significantly different under different conditions (Kruskal–Wallis test, *n* = 5 mice per condition, χ^2^ = 19.86, *p* < 0.001); animals in the 60-min-after-RS group (*p* = 0.030) and those in the 120-min-after-RS group (*p* = 0.032) had a significantly higher number of Zif268-positive cells than Ctrl animals (Steel test; Figure 3H). In each SLEA-zNC, the number of Zif268-positive cells was significantly different among the different experimental conditions (Kruskal-Wallis test; Ctrl, *n* = 10 clusters; 30 min, *n* = 11 clusters; 60 min, *n* = 18 clusters; 120 min, *n* = 15 clusters; or 180 min, *n* = 12 clusters after RS; χ^2^ = 11.30, *p* = 0.023); in the 60-min-after-RS group, there were significantly more cells than in the Ctrl group (Steel test, *p* = 0.044; Figure 3I). The number of SLEA-zNCs was significantly different among the different experimental conditions (Kruskal–Wallis test; *n* = 5 mice per condition; χ^2^ = 13.84, *p* < 0.001); animals in the 60-min-after-RS group had significantly more SLEA-zNCs than Ctrl animals (Steel test, *p* = 0.038; Figure 3J). The results showed that SLEA-zNCs were transiently activated by RS, and the number of Zif268-positive cells peaked at 60 min. In addition, the number of c-Fos + Zif268 double-positive cells per animal was significantly different among the different experimental conditions (Kruskal–Wallis test, *n* = 5 mice per condition, χ^2^ = 19.77, *p* < 0.001); animals in the 180-min-after-RS group had significantly more cells than Ctrl animals (Steel test, *p* = 0.028; Figure 3K). The number of c-Fos + Zif268 double-positive cells per SLEA-zNC was significantly different among experimental conditions (Kruskal–Wallis test; Ctrl, *n* = 10 clusters; 30 min, *n* = 11 clusters; 60 min, *n* = 18 clusters; 120 min, *n* = 15 clusters; or 180 min, *n* = 12 clusters after RS; χ^2^ = 33.23, *p* < 0.001); mice in the 180-min-after-RS group had more double-positive cells than the Ctrl group (*p* < 0.001, Steel test; Figure 3L). Moreover, the number of clusters was significantly different among experimental conditions (Kruskal–Wallis test; *n* = 5 mice per condition; χ^2^ = 12.08, *p* < 0.001); those in the 180-min-after-RS group had more clusters than the Ctrl group (*p* = 0.022, Steel test; Figure 3M). The results showed that c-Fos + Zif268 double-positive cells in SLEA-zNCs peaked at 180 min, confirming different time courses of IEG products’ expression between Zif268 and c-Fos.

**FIGURE 4:**
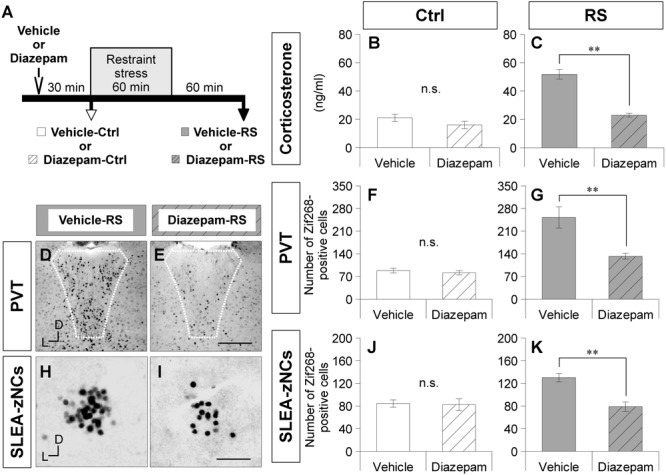
Diazepam treatment suppresses activation of SLEA-zNCs by restraint stress (RS). Time course of experiment with diazepam treatment is shown **(A)**. Vehicle-Ctrl or diazepam-Ctrl indicates pretreatment vehicle or diazepam without RS. Vehicle-RS or diazepam-RS indicates pretreatment vehicle or diazepam with RS. The plasma corticosterone concentrations are shown in the vehicle- and diazepam-Ctrl group, or in the vehicle- and diazepam-RS group [*n* = 6 animals for each group in **(B)** and **(C)**, respectively]. The Zif268-positive cells in the PVT are shown in the vehicle-RS group **(D)** and diazepam-RS group **(E)**. White dotted lines indicate boundary of the PVT. The numbers of the Zif268-positive cells in the PVT are shown in the vehicle- and diazepam-Ctrl group, or in the vehicle- and diazepam-RS group [*n* = 6 animals for each group in **(F,G)**]. The Zif268-positive cells in SLEA-zNCs are shown in the vehicle-RS group **(H)** and diazepam-RS group **(I)**. The numbers of Zif268-positive cells in SLEA-zNCs are shown in the vehicle- and diazepam-Ctrl group, or in the vehicle- and diazepam-RS group [*n* = 6 animals for each group in **(J,K)**]. Data are represented as mean ± SEM. Double asterisks indicate significance (^∗∗^*p* < 0.01, Wilcoxon rank-sum test). n.s. Indicates no significance. D, dorsal; L, lateral; SLEA-zNC, sublenticular extended amygdalar Zif268-expressing neuronal cluster; PVT, paraventricular thalamic nucleus. Scale bars, 250 μm **(D,E)** and 50 μm **(H,I)**.

To verify the effects of RS on SLEA-zNCs, mice were treated with diazepam (a benzodiazepine receptor agonist of GABA-A receptors) or a vehicle solution before RS (Figure 4A). The plasma corticosterone concentrations were not significantly different between the vehicle-Ctrl group and the diazepam-Ctrl group (Wilcoxon rank-sum test, *n* = 6 mice per condition, *p* = 0.383; Figure 4B). Plasma corticosterone concentrations were significantly lower in the diazepam-RS group than in the vehicle-RS group (Wilcoxon rank-sum test, *n* = 6 mice per condition, *p* = 0.002; Figure 4C).

A smaller number of Zif268-positive cells were observed in the PVT of the diazepam-RS than in the vehicle-RS (Figures 4D,E). Although the number of Zif268-positive cells in the PVT was not significantly different between the vehicle-Ctrl group and the diazepam-Ctrl group (Wilcoxon rank-sum test, *n* = 6 mice per condition, *p* = 0.266; Figure 4F), the number of Zif268-positive cells in the PVT was significantly lower in the diazepam-RS group than in the vehicle-RS group (Wilcoxon rank-sum test, *n* = 6 mice per condition, *p* = 0.002; Figure 4G). The results confirmed that pretreatment with diazepam had no effect on Ctrl and inhibited systemic and neuronal effects of RS.

A smaller number of Zif268-positive cells were observed in SLEA-zNCs of the diazepam-RS than the vehicle-RS (Figures 4H,I). Although the number of Zif268-positive cells was not significantly different between the vehicle-Ctrl and the diazepam-Ctrl groups (Wilcoxon rank-sum test; *n* = 6 mice per condition, *p* = 0.844; Figure 4J), the number of Zif268-positive cells was significantly lower in the diazepam-RS group compared to the vehicle-RS group (Wilcoxon rank-sum test, *n* = 6 mice per condition, *p* = 0.004; Figure 4K). The results showed that the diazepam treatment suppressed the activation of SLEA-zNCs by RS, indicating that SLEA-zNCs participates in stress processing circuits.

### Distribution of SLEA-zNCs Between the Right and Left Hemispheres at Different Positions Along the Anteroposterior Axis in Individual Mice

**FIGURE 5:**
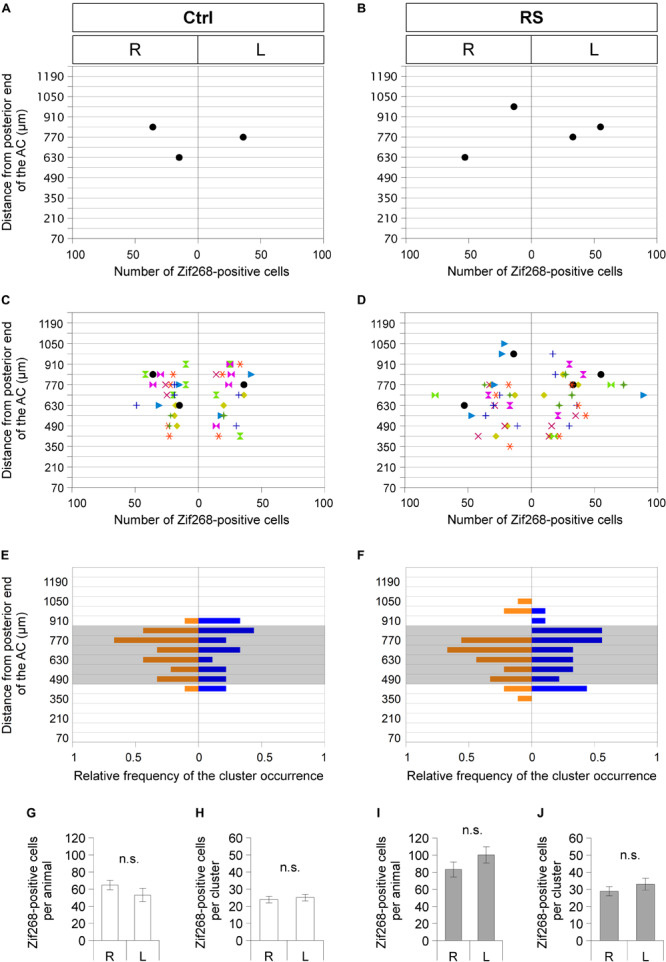
Sublenticular extended amygdalar Zif268/Egr1-expressing neuronal clusters are observed asymmetrically between the right and left hemispheres at different positions along the anteroposterior axis in each mouse. Spatial distribution of SLEA-zNCs and number of neurons in SLEA-zNCs are mapped without restraint stress (RS) (Ctrl) **(A,C,E)** and at 60 min after RS **(B,D,F)**. Distributions of SLEA-zNCs in a representative Ctrl and RS case **(A,B)**. In the vertical axis, distances are shown between the SLEA-zNC and the posterior limbs of the AC. In the horizontal axis, the number of neurons in each SLEA-zNC and their position in the right or left hemisphere are shown. In panels **(C,D)**, distributions of SLEA-zNCs in all Ctrl and RS animals are shown. Same-colored symbols indicate the same animal **(A–D)**; different symbols of different colors indicate different mice. In panels **(E,F)**, in the vertical axis, the relative frequency distribution of SLEA-zNCs is mapped along the anteroposterior axis. In the horizontal axis, relative frequencies of SLEA-zNCs’ appearance in the right (orange) or left (blue) hemisphere are shown; shaded areas indicate middle region between the AC and GP, 490–840 μm posterior to the AC. In Ctrl and RS groups, the total numbers of Zif268-positive cells are not significantly different between the right and left hemispheres [*n* = 9 mice per condition and group (Ctrl and RS)] and in SLEA-zNCs (*n* = 24 clusters for the right and *n* = 19 clusters for the left hemisphere in the Ctrl; *n* = 26 clusters for the right and *n* = 27 clusters for the left hemisphere in the RS) (Wilcoxon rank-sum test) **(G–J)**. Data are represented as mean ± SEM. n.s. Indicates no significance. R, right hemisphere; L, left hemisphere; SLEA-zNC, sublenticular extended amygdalar Zif268-expressing neuronal cluster; AC, anterior commissure; GP, globus pallidus.

Sublenticular extended amygdalar Zif268/Egr1-expressing neuronal clusters were mapped along the anteroposterior axis from the posterior end of the AC to the posterior end of the GP. In a mouse in resting condition without RS (Ctrl), SLEA-zNCs were observed asymmetrically between the right and left hemispheres at different positions along the anteroposterior axis (Figure 5A). This was also true in the case of a mouse with RS activation (Figure 5B). Individual mice revealed that asymmetrical distributions of SLEA-zNCs at different positions along the anteroposterior axis were consistent (*n* = 9 mice; Figures 5C,D). The frequency distribution of SLEA-zNCs was observed; either clusters were found in a resting condition, or, after the activation, they frequently appeared in the middle region (distance between 490 and 840 μm from posterior end of the AC) (Figures 5E,F). Moreover, the frequency distribution showed that SLEA-zNCs appeared equally in both sides of the hemispheres.

In Ctrl animals, the total numbers of Zif268-positive cells in SLEA-zNCs were not significantly different between the right and left hemispheres, and the number of cells per SLEA-zNC was not significantly different between the right and left, either (Figures 5G,H) [*n* = 9 mice; right hemisphere, 64.8 ± 5.5 cells per animal; left hemisphere, 53.1 ± 7.9 cells per animal, *p* = 0.546; right hemisphere, 23.9 ± 1.9 cells per SLEA-zNC (n = 24 clusters); left hemisphere, 25.1 ± 1.9 cells per SLEA-zNC (*n* = 19 clusters), *p* = 0.581, Wilcoxon rank-sum test], indicating that resting activity of SLEA-zNCs in Ctrl was similar between the right and left hemispheres.

In RS animals, the total numbers of Zif268-positive cells were not significantly different between the right and left hemispheres, and the number of the cells per SLEA-zNC was not significantly different between the right and left, either (Figures 5I,J) [*n* = 9 animals; right hemisphere, 83.2 ± 8.7 cells per animal; left hemisphere, 100.2 ± 9.5 cells per animal, *p* = 0.287; right hemisphere, 28.9 ± 2.7 cells per SLEA-zNC (*n* = 26 clusters); left hemisphere, 33.0 ± 3.5 cells per SLEA-zNC (*n* = 27 clusters); *p* = 0.434, Wilcoxon rank-sum test], indicating that activation of SLEA-zNCs in RS animals was similar between the right and left hemispheres. This result indicated that SLEA-zNCs were not lateralized.

Sublenticular extended amygdalar Zif268/Egr1-expressing neuronal clusters were situated at different positions in individual mice; however, it was not clear whether brain coordinates of SLEA-zNCs were different between individual mice and did not vary under the different experimental conditions, or whether the difference in cluster positions was activated under the experimental conditions in the SLEA of each mouse.

### Brain Coordinates of SLEA-zNCs Were Different Between Individuals

**FIGURE 6:**
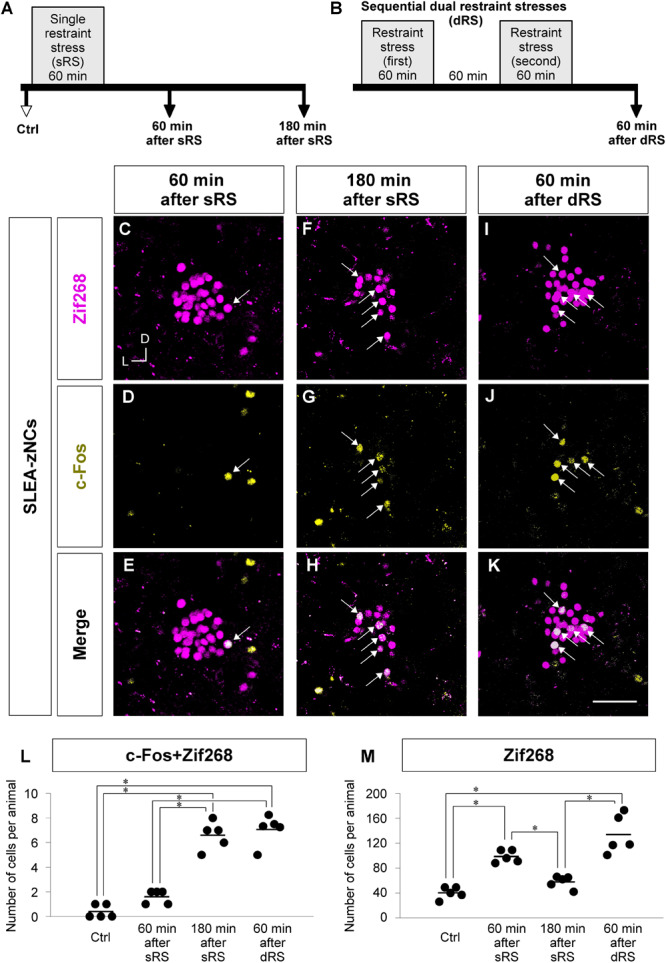
Analyzing c-Fos + Zif268 double-positive and Zif268-positive cells in SLEA-zNCs allows to discriminate whether an animal received a single treatment of restraint stress (sRS) or sequential dual treatment (dRS). Time courses of sRS and dRS are shown **(A,B)**. In sRS, numbers of c-Fos + Zif268 double-positive and Zif268-positive cells are counted at 60 or 180 min after sRS **(A)**. In dRS, they are counted at 60 min after dRS **(B)**. Control (Ctrl) indicates no RS. Zif268-positive cells [magenta in panels **(C,F,I)**], c-Fos-positive cells [yellow in panels **(D,G,J)**], and merged images **(E,H,K)** are shown. Arrows indicate the double-positive cells. The total numbers of c-Fos + Zif268 double-positive cells and Zif268-positive cells per animal in SLEA-zNCs are shown in panels **(L,M)**, respectively. Horizontal lines represent means of values (*n* = 5 mice for each group). Single asterisk indicates significance (^∗^*p* < 0.05, Kruskal-Wallis test followed by Steel-Dwass test). D, dorsal; L, lateral; SLEA-zNC, sublenticular extended amygdalar Zif268-expressing neuronal cluster. Scale bar, 50 μm **(C–K)**.

To distinguish between the two possibilities regarding the individual different positions of SLEA-zNCs, we developed the sequential dRS. Mice were subjected to dRS, single treatment (sRS), and to no treatment (Ctrl) (Figures 6A,B); the effect of each treatment on SLEA-zNCs was estimated in each animal by total numbers of c-Fos + Zif268 double-positive cells and Zif268-positive cells. Dual treatment of RS was useful for discriminating the time course of SLEA-zNCs’ activation because expressions of Zif268 and c-Fos products reflected 60 and 180 min after the stimulation, respectively (Figures 3H–M).

First, we addressed how sRS and dRS differentially affect the number of neurons that constitute SLEA-zNCs and c-Fos-positive neurons among them in individual animals (Figures 6L,M). The total numbers of c-Fos + Zif268 double-positive cells per animal are shown in Figure 6L. The number was significantly different between the experimental conditions (Kruskal–Wallis test, *n* = 5 mice per condition, χ^2^ = 15.01, *p* < 0.001); mice in the 180-min-after-sRS group had significantly more double-positive cells than the Ctrl group (*p* = 0.039) and the 60-min-after-sRS group (*p* = 0.039); mice in the 60-min-after-dRS group had significantly more double-positive cells than the Ctrl group (*p* = 0.040) and the 60-min-after-sRS group (*p* = 0.040) (Steel-Dwass test). The numbers of double-positive cells were not significantly different between the 180-min-after-sRS group and the 60-min-after-dRS group (*p* = 0.720) (Steel-Dwass test; Figure 6L). These results confirmed that SLEA-zNCs’ activation occurring 180 min ago could be discriminated from that occurring 60 min ago by using the c-Fos + Zif268 double expression.

The total numbers of Zif268-positive cells per animal are shown in Figure 6M. The number was significantly different among the experimental conditions (Kruskal–Wallis test, *n* = 5 mice per condition, χ^2^ = 16.83, *p* < 0.001); mice in the 60-min-after-sRS group had more cells than the Ctrl group (*p* = 0.043) and the 180-min-after-sRS group (*p* = 0.044); those in the 60-min-after-dRS group had more cells than the Ctrl group (*p* = 0.044) and the 180-min-after-sRS group (*p* = 0.045) (Steel-Dwass test), confirming that SLEA-zNCs’ activation 60 min ago was discriminated from that occurring 180 min ago by using the Zif268 expression. Thus, analyzing c-Fos + Zif268 double-positive and Zif268-positive cells in each animal allowed to discriminate whether the animal received, or not, dRS (60 min after dRS) or sRS (180 min after sRS).

**FIGURE 7:**
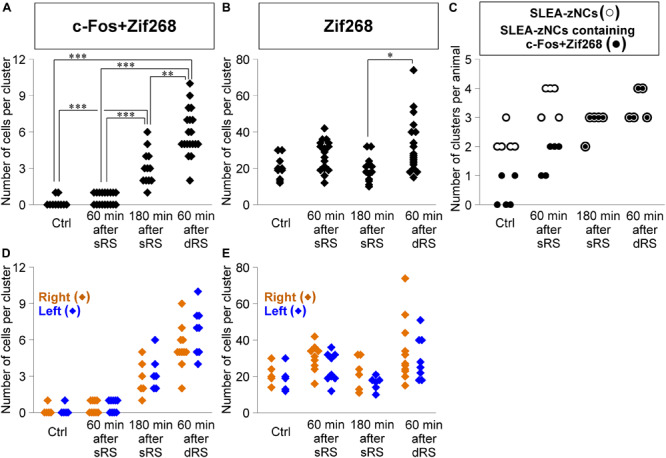
Sublenticular extended amygdalar Zif268/Egr1-expressing neuronal clusters are activated by both the first and second stimulation in the sequential dual treatment of restraint stresses (dRS). The numbers of c-Fos + Zif268 double-positive cells are compared between Ctrl, at 60 min, at 180 min after the single treatment of restraint stress (sRS), and at 60 min after dRS **(A)** (n = 10, 18, 12, 19 clusters for each group, respectively). The numbers of Zif268-positive cells **(B)**. The numbers of SLEA-zNCs and those containing c-Fos + Zif268 double-positive cells are shown for each mouse; SLEA-zNCs of an individual mouse are on the same vertical line **(C)**. The number of SLEA-zNCs in each animal is depicted as open circles; the number of SLEA-zNCs containing c-Fos + Zif268 double-positive cells in each animal is depicted as closed circles. Moreover, filled circles with a white perimeter indicate that c-Fos + Zif268 double-positive cells are contained in all SLEA-zNCs in all animals in 180 min after sRS and 60 min after dRS. The numbers of c-Fos + Zif268 double-positive cells in SLEA-zNCs are shown in the right and left hemispheres **(D)**. The numbers of Zif268-positive cells in SLEA-zNCs are shown in the right and left hemispheres **(E)**. The right SLEA-zNCs are indicated as orange diamonds, and the left as blue. Values are compared with the Kruskal-Wallis test followed by the Steel-Dwass test. Data are represented as mean ± SEM. Single, double, and triple asterisks indicate significances (^∗^*p* < 0.05, ^∗∗^*p* < 0.01, ^∗∗∗^*p* < 0.001). SLEA-zNC, sublenticular extended amygdalar Zif268-expressing neuronal cluster; sRS, a single treatment of restraint stress; dRS, sequential dual treatment of restraint stress.

Second, we addressed how dRS differentially affected the numbers of c-Fos + Zif268 double-positive cells and Zif268-positive cells in each cluster, by identifying which clusters are activated by the first and/or second stimulation in dRS (Figures 7A,B). The number of c-Fos + Zif268 double-positive cells at 180 min after sRS (range, 1–6 cells; 3.1 ± 0.3) was significantly different from those in Ctrl (range, 0–1 cell; 0.2 ± 0.1; *p* < 0.001) and at 60 min after sRS (range, 0–1 cell, 0.5 ± 0.1, *p* < 0.001) (χ^2^ = 48.258, Kruskal–Wallis test followed by Steel-Dwass test), confirming that the number of the double-positive cells reflected activation occurring 180 min ago. At 60 min after dRS, the number of the double-positive cells was unimodally distributed within the range of 2–10 cells (5.9 ± 0.4), which was significantly different from those in Ctrl (*p* < 0.001) and at 60 min after sRS (*p* < 0.001) (Figure 7A). Thus, analysis of c-Fos + Zif268 double-positive cells showed that all SLEA-zNCs were activated by the first stimulation in dRS.

Moreover, at 60 min after dRS, the number of Zif268-positive cells was distributed within the range of 15–74 cells (32.4 ± 3.3), which was not different from that at 60 min after sRS (range, 12–42 cells; 26.0 ± 2.3; *p* = 0.93). However, that was significantly larger than that at 180 min after sRS (range, 10–32 cells; 19.3 ± 1.9; *p* < 0.05; χ^2^ = 13.394) (Figure 7B). Analysis of Zif268-positive cells indicated that SLEA-zNCs responded to the second stimulation in dRS.

The dRS analysis per cluster indicated that SLEA-zNCs responded to the first and second stimulation. These results raised a question whether all SLEA-zNCs responded to both the first and second stimulations. To address this, we analyzed the number of SLEA-zNCs containing c-Fos + Zif268 double-positive cells in each mouse. All SLEA-zNCs from all mice examined contained c-Fos + Zif268 double-positive cells 60 min after dRS, which was the same result as that at 180 min after sRS. However, this was different from that of Ctrl and at 60 min after sRS, in which only a part of SLEA-zNCs contained the double-positive cells. These results indicate that, in the dRS condition, all SLEA-zNCs responded to the first stimulation with c-Fos expression; however, no SLEA-zNCs responded exclusively to the second stimulation (Figure 7C). Total numbers of SLEA-zNCs were significantly different between Ctrl and 60 min after sRS (*p* < 0.05) and between Ctrl and 60 min after dRS (*p* < 0.05) (χ^2^ = 11.43, Kruskal–Wallis test followed by Steel-Dwass test). Taken together, these results indicate that the same SLEA-zNCs responded to both the first and second stimulation in dRS, and this applied for all SLEA-zNCs.

In addition, the numbers of c-Fos + Zif268 double-positive cells and Zif268-positive cells in each SLEA-zNC were compared between the right and left hemispheres. At 60 min after dRS, the number of c-Fos + Zif268 double-positive cells in each SLEA-zNC was not significantly different between the right and left hemispheres (right hemisphere, *n* = 11 clusters; left hemisphere, *n* = 8 clusters; *p* = 0.42, Wilcoxon rank-sum test) (Figure 7D); this also applied for the number of Zif268-positive cells (right hemisphere, *n* = 11 clusters; left hemisphere, *n* = 8 clusters; *p* = 0.45, Wilcoxon rank-sum test) (Figure 7E). The distributions of the double-positive and single-positive cells were intermingled between the right and left, and no spatial tendency of the SLEA-zNCs’ activation between the right and left was observed in dRS. The results indicated that SLEA-zNCs responded to the stimulations with no bias between the right and left hemispheres, confirming that all SLEA-zNCs responded to both the first and second stimulation of dRS.

**FIGURE 8:**
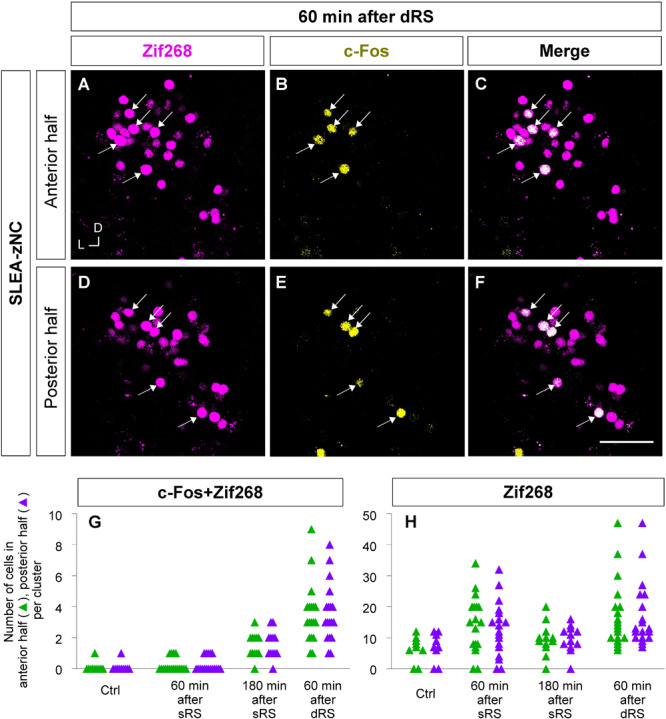
Anterior and posterior halves of SLEA-zNCs responded to the first and second stimulation of the sequential treatment of restraint stresses (dRS). Zif268- (magenta), c-Fos- (yellow), and merged images of anterior halves of SLEA-zNC are shown at 60 min after dRS [**(A–C)**, respectively], and the posterior halves are shown **(D–F)**. Arrows indicate double-positive cells. The numbers of c-Fos + Zif268 double-positive cells are shown in the anterior and posterior halves of each SLEA-zNC **(G)**. The numbers of Zif268-positive cells are shown in the anterior and posterior halves of each SLEA-zNC **(H)**. Those in the anterior halves of SLEA-zNC are indicated as green triangles and in the posterior as purple. SLEA-zNC, sublenticular extended amygdalar Zif268-expressing neuronal cluster; sRS, a single treatment of restraint stress; dRS, sequential dual treatment of restraint stress.

Furthermore, to examine the spatial pattern of neuronal activation within each SLEA-zNC, optical sections of every SLEA-zNC were divided into anterior and posterior halves; then, c-Fos + Zif268 double-positive cells and Zif268-positive cells were counted in each half (Figures 8A–F). At 60 min after dRS, the number of c-Fos + Zif268 double-positive cells in each SLEA-zNC was not significantly different between the anterior and posterior halves (anterior halves of *n* = 19 clusters; posterior halves of *n* = 19 clusters; *p* = 0.97, Wilcoxon rank-sum test), indicating that distribution of the double-positive cells was intermingled between the anterior and posterior halves with no specific spatial distribution (Figure 8G).

The number of Zif268-positive cells was not significantly different between the anterior and posterior halves (anterior halves of *n* = 19 clusters; posterior halves of *n* = 19 clusters, *p* = 0.96; five mice for each condition; Wilcoxon rank-sum test), indicating that Zif268-positive cells showed no specific spatial distribution between the anterior and posterior halves (Figure 8H). Because the numbers of c-Fos + Zif268 double-positive cells and Zif268-positive cells reflect activation by the first and second stimulation, respectively, the results indicated that SLEA-zNCs responded to the stimulations with no bias between the anterior and posterior halves.

The dRS experiment demonstrated that (1) the same SLEA-zNCs responded to the first and second stimulation of dRS, (2) SLEA-zNCs responded to the stimulations with no bias between the right and left hemispheres, and (3) SLEA-zNCs responded to the stimulations with no bias between the anterior and posterior halves. Moreover, and (4) it is highly probable that all SLEA-zNCs responded to both the first and second stimulation of dRS. Therefore, brain coordinates of SLEA-zNCs were thought to be different between individuals.

## Discussion

We found novel neuronal clusters in SLEA, SLEA-zNCs, responding to stress stimuli. The RS induced Zif268 expression in SLEA-zNCs, and its induction was suppressed by antistress treatment with diazepam (Figures 3, 4; Umemoto et al., 1997; Sztainberg et al., 2011; Kovács et al., 2018), indicating that SLEA-zNCs participate in stress processing. Sublenticular extended amygdalar Zif268/Egr1-expressing neuronal clusters were not distinguishable by their cytoarchitecture but by expression of Zif268 (Figures 2A–C). Sublenticular extended amygdalar Zif268/Egr1-expressing neuronal clusters were reproducibly observed in all mice examined from two different strains [C57BL/6J and ICR (data not shown)].

Sublenticular extended amygdalar Zif268/Egr1-expressing neuronal clusters were found in the area between the EA, GP, and IPAC. Analysis of cell-type markers showed that the SLEA-zNC consists of heterogeneous types of cells, although their proportion is comparable in each cluster. Sublenticular extended amygdalar Zif268/Egr1-expressing neuronal clusters consisted mostly of GABAergic neurons and were different from known types of neurons in the EA and GP; thus, SLEA-zNC is a novel population of neuronal cluster. In the GP, GABAergic neurons are classified into two categories: prototypic neurons, mostly expressing PV, and arkypallidal neurons, exclusively expressing PENK and FoxP2 (Mallet et al., 2012; Abdi et al., 2015; Dodson et al., 2015; Hernández et al., 2015). Although the presence of a small percentage of PENK- and FoxP2-positive cells suggests that SLEA-zNCs partially share molecular features with arkypallidal neurons, their expression profile indicates that they are not likely to be prototypic or arkypallidal neurons (Figure 2K). The percentages of CB- and PENK-positive neurons indicated some degree of overlap between CB and PENK expression in the SLEA-zNCs, although it has been reported that CB-positive cells express enkephalin less frequently, for example, in the superficial dorsal horn of spinal cord (Yoshida et al., 1990). The colocalization of CB/PENK and the expression of PENK/FoxP2 in SLEA-zNCs, along with their functional significance, will be the subject of future studies. It is known that EA neurons coexpress GAD65 and ChAT (Granger et al., 2016); however, SLEA-zNCs consisted of a few cholinergic neurons (Figure 2K).

Sublenticular extended amygdalar Zif268/Egr1-expressing neuronal clusters were located medially to the IPAC. The IPAC is activated by social defeat stress and is regulated by the dopamine D1 receptor (Numa et al., 2019) and revealed a high density of Zif268- (Figure 1) and CB-positive cells (data not shown); thus, the IPAC is distinct from the SLEA-zNC area. Moreover, neurons in the IPAC express Arc (activity-regulated cytoskeleton associated protein) and ChAT (Tanaka et al., 2019); however, neurons in SLEA-zNCs did not express Arc (data not shown), and only few expressed ChAT (Figure 2). Therefore, although both SLEA-zNCs and IPAC are activated by stress, they are considered to be different.

The SLEA is thought to be the special region that receives projections from the central nucleus of amygdala (CeA) and the dorsolateral division of the bed nucleus of the stria terminalis (dlBNST; Gastard et al., 2002). Sublenticular extended amygdalar Zif268/Egr1-expressing neuronal clusters responded to the stress stimuli, which is consistent with the fact that the SLEA receives outputs from the CeA/dlBNST. When a retrograde tracer, the cholera toxin subunit B, was injected into the cortex, labeled neurons were observed in the ChAT-positive cholinergic neurons surrounding SLEA-zNCs, but not within SLEA-zNCs. In addition, no retrogradely labeled neurons were found in SLEA-zNCs from the caudate-putamen, mediodorsal thalamic nucleus, or lateral habenula (data not shown). It is possible that the neurons in SLEA-zNCs are involved in local circuits. Sublenticular extended amygdalar Zif268/Egr1-expressing neuronal clusters are thought to be functional units in the stress processing acting as novel “islands of activation,” because they expressed IEGs on the same time schedule (Guzowski et al., 2005; Ohkawa et al., 2015). Neuroanatomical organization of SLEA-zNC between the CeA/dlBNST and corticopetal projections of cholinergic neurons remains to be elucidated further.

Sublenticular extended amygdalar Zif268/Egr1-expressing neuronal clusters were situated in different positions in individual mice. The mapping method used, although accurate, may have produced an error of position along the anteroposterior axis within the thickness of the sections (70 μm). However, SLEA-zNCs were found far beyond the adjacent sections in each mouse; thus, the results confirmed that they were distributed asymmetrically in the right and left hemispheres (Figures 5A–D). Although SLEA-zNCs were in different positions along the anteroposterior axis, their positions were not random; they frequently appeared in the middle region, in between the AC and GP, which is the anterior end of the hippocampus and the basolateral amygdala (in close proximity to Bregma −0.83 mm) (Paxinos and Franklin, 2013).

Two possibilities exist regarding how different positions of SLEA-zNCs occurred. The first is that geographical positions of SLEA-zNCs were different between individuals, and their positions would not have varied among different experimental conditions in the same mouse. The second is that, in neuronal populations common in all mice, the positions that were activated varied depending on the experimental conditions in each mouse, forming SLEA-zNCs. To distinguish between these two possibilities, we developed the sequential dRS, by taking advantages of IEGs’ activation with different time schedules (Guzowski et al., 2001; Zangenehpour and Chaudhuri, 2002; Gallo et al., 2018). In animal level, different time peaks of Zif268 and c-Fos expression after a stimulation allowed to discriminate whether the animal received dRS (60 min after dRS) or sRS (180 min after sRS) (Figures 6L,M). In terms of clusters, this analysis enabled us to confirm that all the SLEA-zNCs are activated by both the first or second stimulations in dRS. Analyzing dRS by c-Fos + Zif268 double-positive cells showed that SLEA-zNCs were activated by the first stimulation, but none were activated exclusively by the second stimulation (Figure 7A). In addition, analyzing by Zif268-positive cells indicated that SLEA-zNCs responded to the second stimulation in dRS. In animal level, the response of SLEA-zNCs to the second stimulation in dRS was obvious in Zif268-positive cells, comparing 60 min after dRS with Ctrl and 180 min after sRS (Figure 6M) although, in cluster level, this tendency was less remarkable than in animal level. It was likely because considerable number of Zif268-positive cells was observed in condition with no stimulation (spontaneous expression) and Zif268 showed less clear response after the stimulation than c-Fos (Figure 7B). However, all SLEA-zNCs contained c-Fos + Zif268 double-positive cells 60 min after dRS (this is the same as 180 min after the first stimulation and 60 min after the second stimulation), indicating that they responded to the first stimulation; moreover, the number of SLEA-zNCs increased 60 min after dRS, indicating that they responded to the second stimulation (Figure 7C). These results indicate that the same SLEA-zNCs responded to both the first and second stimulation in dRS. This also applied in the case of all SLEA-zNCs. In addition, SLEA-zNCs responded to the stimulations with no bias between the right and left hemispheres, confirming that all SLEA-zNCs responded to the first and second stimulation of dRS (Figures 7D,E). Moreover, SLEA-zNCs responded to the stimulations with no bias between the anterior and posterior halves (Figures 8G,H). All these results support that the geographical positions of SLEA-zNCs are different between individuals and invariable under different experimental conditions in each mouse. Although individual differences in the amygdala within the same species have been reported (Lomnicki, 1978; Schaefer et al., 2006; Yang et al., 2008; Stamps et al., 2012; Armario and Nadal, 2013; Rice et al., 2014), this is the first indication of individual differences of SLEA-zNC positions in the BF.

In general, the principle of dRS is applicable for discriminating the time course of neuronal activation after any sequential dual stimulations. There are also methods with higher time resolution than dRS. One of these methods relies on measurement of Arc, which shows an expression peak 30 min after stimulation (Guzowski et al., 1999, 2001). However, Arc was not immunohistochemically detected in SLEA-zNCs (data not shown). Another method is cellular compartment analysis of temporal activity by fluorescence *in situ* hybridization (catFISH; Guzowski et al., 2005; Lin et al., 2011). However, the catFISH method was not practical in this study because low accumulation of *zif268/egr1* mRNA made it difficult to identify SLEA-zNCs (data not shown). Therefore, SLEA-zNCs in each mouse are thought to be activated together after the stimulation in a temporal resolution of approximately 60 min.

Arrangements in different positions of SLEA-zNCs are likely to cause variances in their circuits’ length, resulting in possible variations in the processing speed or efficiency among individuals. The functional influence of different cluster positions is intriguing because SLEA-zNCs are thought to participate in the circuits of stress processing closely related to animal survival.

Many vertebrates have asymmetry in the nervous system (Concha and Wilson, 2001). There are at least two known types of circuit asymmetry (Ichijo et al., 2017). The first type of circuit asymmetry is that of structurally asymmetrical circuits with lateralized function causing lateralized behavior in sensory and motor systems (Rogers and Anson, 1979; Evans et al., 1993; Rogers, 2000). In the second type of circuit asymmetry, it is hypothesized that the structurally asymmetrical circuits are related to binary opposite behaviors in functional incompatibility (Concha et al., 2003; Aizawa et al., 2005; Amo et al., 2010; Chou et al., 2016; Ichijo et al., 2017). However, SLEA-zNCs were not functionally lateralized because the total numbers of activated neurons in SLEA-zNCs were similar between the right and left hemispheres, even though they were distributed asymmetrically between the EA and GP (Figures 5G–J). Because the EA and GP are derived from different lineages of medial, lateral, and/or caudal ganglionic eminence (Marín and Rubenstein, 2001; Flames et al., 2007; Anastasiades and Butt, 2011), it is possible that SLEA-zNCs form in different positions under the influence of stochastic interaction between intermingling cells of different lineages.

## Conclusion

We have identified novel stress-related SLEA-zNCs that are functionally discernible by Zif268 expression and are thought to be functional units of “islands of activation.” Sublenticular extended amygdalar Zif268/Egr1-expressing neuronal clusters are located in different positions in individual mice; such individual differences have not been previously reported.

## Data Availability Statement

The raw data supporting the conclusions of this article will be made available by the authors, without undue reservation, to any qualified researcher.

## Ethics Statement

All experimental protocols were performed in accordance with the guidelines for care and use of laboratory animals approved by the University of Toyama (Sugitani, Toyama, Japan) and the National Institutes of Health’s Guide for the Care and Use of Laboratory Animals, and approved by the Ethics Committee for Animal Experiments at the University of Toyama (License number: A2016MED-38).

## Author Contributions

MK, TN, and HI designed the experiments. HI conceived and supervised the project. MK performed the experiments and analysis. TN contributed to data collection. MS contributed to data collection of R26R-H2B-mCherry Cre-reporter mice. MK and HI wrote the manuscript. All authors approved the final version of the manuscript and agreed to be accountable for all aspects of the work in ensuring that questions related to the accuracy or integrity of any part of the work are appropriately investigated and resolved.

## Conflict of Interest

The authors declare that the research was conducted in the absence of any commercial or financial relationships that could be construed as a potential conflict of interest.

## Footnotes

1 https://imagej.nih.gov/ij/

2 https://www.r-project.org/
